# Public Perceptions of COVID-19 in Australia: Perceived Risk, Knowledge, Health-Protective Behaviors, and Vaccine Intentions

**DOI:** 10.3389/fpsyg.2020.551004

**Published:** 2020-09-30

**Authors:** Kate Faasse, Jill Newby

**Affiliations:** ^1^School of Psychology, UNSW Sydney, Sydney, NSW, Australia; ^2^Black Dog Institute, UNSW Sydney, Sydney, NSW, Australia

**Keywords:** COVID-19, emerging infectious disease, health behaviors, perceived risk, worry, knowledge, uncertainty, misinformation

## Abstract

Widespread and sustained engagement with health-protective behaviors (i.e., hygiene and distancing) is critical to successfully managing the COVID-19 pandemic. Evidence from previous emerging infectious disease outbreaks points to the role of perceived risk, worry, media coverage, and knowledge in shaping engagement with health-protective behaviors and vaccination intentions. The aim of the current study was to examine the role of these factors in predicting recommended health-protective behaviors early in the pandemic. A secondary aim was to assess uncertainty and misconceptions about COVID-19. An online survey of 2,174 Australian residents was completed between March 2 and 9, 2020, at an early stage of the COVID-19 outbreak in Australia. Results revealed that two-thirds of respondents were at least moderately worried about a widespread COVID-19 outbreak. Worry about the outbreak and closely following media coverage were consistent predictors of greater engagement with health-protective behaviors and higher vaccination intentions. Uncertainty and misconceptions about COVID-19 were common, including uncertainty about whether people are likely to have natural or existing immunity to the virus. There was also uncertainty around whether specific home remedies (e.g., vitamins and saline rinses) would offer protection and whether the virus was human-made and deliberately released. Such misconceptions are likely to cause concern for members of the public. The findings also highlight psychological and demographic factors associated with lower engagement with health-protective behaviors, including male gender, younger age, and low levels of worry about the outbreak. These findings offer potential pathways and targets for interventions to encourage health-protective behaviors. The results relating to uncertainty and misconceptions about COVID-19 point to areas that could be usefully targeted by public information campaigns.

## Introduction

On December 31, 2019, the first report of a “pneumonia of unknown cause” was made to the World Health Organization (WHO) Country Office (World Health Organization, 2020c). The report came from Wuhan, China. On January 10, 2020, WHO issued its first guidance on the “novel coronavirus,” with similarities to other coronaviruses such as SARS (Severe Acute Respiratory Syndrome) and MERS (Middle East Respiratory Syndrome). By the end of January 2020, the novel coronavirus had spread to countries around the world, and the outbreak was declared a Public Emergency of International Concern. The first cases of COVID-19 in Australia were identified on January 25, 2020 (Minister for Health, 2020). As of March 7, there were 63 confirmed cases, including two deaths (Australian Government Department of Health, 2020b).

Public engagement with health-protective behaviors, including social distancing and hygiene behaviors, has been highlighted as one of the most important strategies for reducing the transmission of COVID-19 (Bonell et al., 2020). Social distancing refers to minimizing the number of times people come into close contact with one another. Hygiene behaviors are those aimed at cleaning hands, surfaces, or objects that may have come into contact with potentially infectious respiratory droplets (Michie et al., 2020). Understanding the cognitive and affective factors that predict engagement with these health-protective behaviors can help inform public health strategies to encourage people to increase and sustain these behaviors.

A number of cognitive factors contribute to engagement with health-protective behaviors during disease outbreaks. Perceived risk, or perceived susceptibility to a threat, has emerged as a consistent predictor of such behaviors (Weinstein, 1988; Petrie et al., 2016). For example, higher perceived likelihood and severity of influenza A/H1N1 (swine flu), influenza H5N1 (bird flu), and SARS were associated with increased heath-protective behaviors in general population samples (Tang and Wong, 2003; Lau et al., 2007; Rubin et al., 2009). Other cognitive factors identified include more accurate knowledge about how a virus is spread (Petrie et al., 2016) and the perception that behaviors will be effective in reducing the risk of infection (Bish and Michie, 2010).

Affective factors also appear to play a role in increasing health-protective behaviors (Slovic et al., 2007). The COVID-19 pandemic has generated substantial public anxiety, uncertainty, and distrust (Asmundson and Taylor, 2020). Both heightened anxiety and trust in information provided by authorities have been shown to predict health-protective behaviors during infectious disease outbreaks (Bish and Michie, 2010).

Substantial media coverage and misinformation have been generated by the COVID-19 pandemic (Asmundson and Taylor, 2020). Media coverage about a health threat can heighten both perceived risk and anxiety (Paek and Hove, 2017). Misinformation and conspiracy theories regarding COVID-19 are also widespread and evolving (Wikipedia, 2020; World Health Organization, 2020a). Such misinformation can have lasting impacts, including reduced engagement with health-protective behaviors including vaccination, once it becomes available (Zimet et al., 2013).

The current study investigated the Australian public’s perception of risk (i.e., likelihood and severity) and worry about COVID-19, viewing of media coverage, accuracy of current knowledge (and conversely, misinformation) about the virus, and health-protective behaviors. Insight into how perceptions of emerging infectious diseases influence the adoption of health-protective behaviors is important in understanding the potential health, social, and economic impact of such outbreaks and may contribute to targeting public health messaging to encourage appropriate health behaviors.

## Materials and Methods

### Recruitment

Members of the Australian general population were recruited for the online survey by the use of Facebook advertisements. Advertisements were targeted at all users with current country of residence listed as Australia and age listed as 18 or above. Users meeting these criteria were shown the advertisement on their Facebook page “timeline.” In addition, the advertisement was posted on the timeline of one university webpage, so that those individuals who followed this page could view the post on their timeline. Facebook users who viewed the advertisement were able to click on an embedded link that took them to the survey (hosted on Qualtrics). Advertising and data collection ran for 7 days from 4 pm Monday, March 2, to 4 pm Monday, March 9, 2020. In total, the ad was displayed to 66,210 individual accounts, with 4,353 clicks. Each response came from a unique Internet Protocol (IP) address, indicating that each response came from a separate device.

### Ethical Approval

The study was approved by the UNSW Human Research Ethics Advisory Panel (File 3309), and all participants provided electronic informed consent to participate.

### COVID-19 in Australia

During the week that the study was conducted, the COVID-19 virus was already in Australia, but infections were limited and were predominantly cases where individuals had contracted the virus overseas (Australian Government Department of Health, 2020a; Worldometer, 2020). At the end of day 1 (March 2), there were 33 confirmed cases; the first death from the virus occurred on this day, as did the first reported community transmissions. This number had risen to 93 by March 9, with three deaths and 18 cases that were likely to be community transmissions (no history of recent travel).

### Participants

In total, 3,086 people viewed the participant information statement and consent form. Of these, 854 either did not consent or completed only some of the survey questions before discontinuing, and 2,232 submitted the survey. Nine responses were excluded because participants reported that they did not live in Australia, and 49 responses were incomplete (48 missing demographic information, one with less than half of all responses completed). This resulted in a final sample of 2,174 participants.

### Measures

See Table 1 for survey questions and response options.

**TABLE 1:** Survey questions and response options.

**Survey question**	**Response options**
**Information**	
How closely have you been following news about the recent outbreak of COVID-19 coronavirus?	11-point scale from 0, *not at all*, to 10, *very closely*
How have you been getting information about the COVID-19 coronavirus outbreak?	Select all that apply: news media, social media, official government websites, family members, friends or colleagues, none of the above, other (text entry)
To what extent do you believe that scientists and other medical and health experts understand the COVID-19 coronavirus?	11-point scale from 0, *don’t understand at all*, to 10, *understand very clearly*
**Perceived risk and worry**	
How concerned or worried are you that there will be a large outbreak of COVID-19 coronavirus in Australia within the next 12 months?	5-point scale: not at all concerned, a little concerned, moderately concerned, very concerned, extremely concerned
How likely do you think it is that there will be an outbreak of COVID-19 coronavirus in Australia?	Visual analogue scale (VAS) from 0, *not at all likely*, to 100, *extremely likely*
If there is an outbreak of COVID-19 coronavirus in Australia, how likely is it that you, personally, will catch the coronavirus?	VAS from 0, *not at all likely*, to 100, *extremely likely*
If there was a COVID-19 coronavirus outbreak in Australia, how much could you personally do to protect yourself from catching the virus?	VAS from 0, *couldn’t do anything*, to 100, *could do a lot*
If you did catch COVID-19 coronavirus, how serious do you think your symptoms would be?	6-point scale: no symptoms, mild symptoms, moderate symptoms, severe symptoms, severe symptoms requiring hospitalization, and severe symptoms leading to death
**Knowledge**	
General virus knowledge, symptoms knowledge, and transmission knowledge	See Tables 3, 4
To minimize the transmission of the COVID-19 coronavirus, who should be wearing a face mask?	Four response options: sick people—to stop them spreading the virus, healthy people—to prevent infection, everyone, no one
To your knowledge, approximately what percentage of people who have been infected with coronavirus (COVID-19) have died from the virus?	VAS from 0% to 100%
**Health-protective behaviors**	
Distancing and hygiene behaviors	See Table 5
If there was a safe and effective vaccine developed for the COVID-19 coronavirus, how likely is it that you would choose to have this vaccination?	5-point scale: would definitely not get the vaccine, would probably not get the vaccine, unsure if I would get the vaccine or not, would probably get the vaccine, would definitely get the vaccine

#### Information

Participants were asked how closely they had been following news about the outbreak, sources of information about the outbreak, and the extent to which they believe that scientists and other medical and health experts understand COVID-19 (to assess perceived scientific understanding).

#### Perceived Risk and Worry

Participants were asked five questions relating to their perceived risk and worry about COVID-19. The first question assessed how concerned or worried respondents were feeling about the possibility of a widespread outbreak in Australia (i.e., the virus spreading from person to person more like a typical cold or flu virus). Perceived likelihood of an outbreak, perceived likelihood of the individual catching the virus if there was an outbreak, perceived behavioral control, and perceived severity were also assessed.

#### Knowledge

To assess knowledge (and possible misinformation), participants were asked to respond to a series of statements about the COVID-19 coronavirus and whether (to the best of their knowledge) these statements were true or false or they were unsure of the answer. See Table 3 for items and their correct answers (based on the state of knowledge at the time of the study). Correctly answered items were summed to generate a general virus knowledge subscale score.

Participants were asked to identify the most common symptoms of COVID-19 infection (see Table 4), based on information provided to the Australian public at the time: fever, cough, sore throat, and shortness of breath (Australian Government Department of Health, 2020c). More recent information includes fatigue or tiredness, which were not included in the survey. Three uncommon symptoms were included: diarrhea, vomiting, and nausea (Guan et al., 2020). The number of correctly answered items was summed to generate a symptoms knowledge subscale score.

Transmission knowledge items asked about the ways the virus can potentially be spread (see Table 4), including droplets spread through coughing or sneezing, touching or shaking hands with someone who is infected, and touching surfaces that have come into contact with the virus. Three other sources, which did not appear to be transmission mechanisms, were also included: water, mosquitoes, and airborne spread (Centers for Disease Control and Prevention, 2020; World Health Organization, 2020a,b). As above, the number of correctly answered items was summed to generate a transmission knowledge subscale score.

One item assessed knowledge of recommended face mask use, with advice to the public at that time being that only people who were sick should be wearing masks to stop them spreading the virus (Australian Government Department of Health, 2020c). Another item assessed knowledge of the approximate mortality rate, which at the time was estimated to be 3.4% (World Health Organization, 2020d). Responses were deemed correct if they were between 1 and 5%. A total COVID-19 knowledge score was calculated as the number of correct responses to all items assessing various aspects of knowledge about COVID-19, potentially ranging from 0 to 34.

#### Health-Protective Behaviors

To assess distancing and hygiene behaviors, participants were asked whether they had engaged in 13 behaviors during the previous month (see Table 4). Response options were *yes*, *no*, *unsure*, and *not applicable*. Items were generated based on previous research (Rubin et al., 2009; Bults et al., 2015; Petrie et al., 2016; Simpson et al., 2019) and recommended behaviors (Australian Government Department of Health, 2020c). Health-protective behavior sum scores (number of “yes” responses) were calculated, with possible scores ranging from 0 to 13.

Participants were asked to complete a single item asking about how likely it is that they would choose to have a COVID-19 vaccination. Responses were scored such that higher scores indicated higher vaccine intentions.

#### Demographics and Health Information

Information was collected on participants’ age group, gender, ethnicity, highest level of education, and region of residence within Australia (see Table 2). Participants were also asked to complete three questions relating to their health. First was a single-item measure assessing their self-rated heath (Idler and Benyamini, 1997), with responses on a five-point scale from *poor* to *excellent*. Second was an item assessing whether they had received a flu vaccine in the previous year (*yes*, *no*, *unsure*). For the purposes of analysis, *no* and *unsure* responses were combined to form a dichotomous measure. Finally, participants were asked whether they, or any family members or friends, had caught COVID-19 (*yes*, *no*, and *unsure*). Only nine respondents said “yes” to this question, and these responses were included in the analysis.

**TABLE 2:** Demographic characteristics of the sample with number (percentage) of respondents.

**Demographic variables**	**Total *N* (%)**
**Gender**	
Male	503 (23.1)
Female	1635 (75.2)
Non-binary, different identity, or prefer not to say	36 (1.7)
**State**	
New South Wales	934 (43.0)
Victoria	312 (14.4)
Queensland	387 (17.8)
South Australia	122 (5.6)
Western Australia	261 (12.0)
Tasmania	87 (4.0)
Australian Capital Territory	52 (2.4)
Northern Territory	19 (0.9)
**Age group**	
18–29	489 (22.5)
30–49	857 (39.4)
50–59	487 (22.4)
60+	303 (13.9)
Not stated	38 (1.7)
**Ethnicity**	
Caucasian (White/European)	1,639 (75.4)
Australian Aboriginal or Torres Strait Islander	178 (8.2)
Asian	173 (8.0)
Other or prefer not to say	184 (8.5)
**Highest Education**	
High school only: completed (Year 12) or not completed (Year 11 or below)	534 (24.6)
Trade certificate, diploma, or advance diploma	528 (24.3)
Bachelor’s degree	562 (25.9)
Graduate diploma, graduate certificate, or postgraduate degree	543 (25.0)
Not stated	7 (0.3)

## Results

### Demographics

Demographic characteristics of the sample can be seen in Table 2. A large proportion of respondents were from the state of New South Wales (NSW).

#### Health-Related Characteristics

Respondents’ mean self-rated health was 3.21 (*SD* = 0.98). The majority of participants rated their health as good (38.7%) or very good (29.5%). Approximately half of the sample (52.9%) reported having had a flu vaccine in the past year. Only nine respondents (0.4%) reported that they themselves, or their friends or family, had caught COVID-19. The majority had not (95.3%).

### Information

Participants reported following news about COVID-19 closely (*M* = 7.3, *SD* = 2.1). Information about COVID-19 came from the news media (85.2%), official government websites (72.2%), social media (68.5%), colleagues or friends (22.7%), and family members (22.7%). Only 0.3% of respondents reported not getting information from any of these sources. Perceived scientific understanding was moderate (*M* = 6.1, *SD* = 2.0).

### Perceived Risk

Concern about the possibility of a widespread outbreak in Australia was moderate (*M* = 3.2, *SD* = 1.1; scale from 1 to 5). A small proportion reported being not at all concerned (6.1%), while 24% reported being a little concerned, 31.1% were moderately concerned, 21.7% were very concerned, and 14.9% were extremely concerned. Respondents’ ratings of the perceived likelihood of an outbreak of COVID-19 in Australia were relatively high (*M* = 71.8, *SD* = 24.9; scale from 0 to 100), and perceived likelihood that they would catch the virus in the case of an outbreak was moderate (*M* = 54.9, *SD* = 24.7). Perceived behavioral control was relatively high (*M* = 68.2, *SD* = 21.6).

With regard to perceived severity of symptoms in the case of infection, only 0.3% of respondents indicated that they would experience no symptoms; mild (27.5%) and moderate (46.7%) symptoms were most commonly anticipated. One in four respondents perceived the illness severity to be high, with 14.1% indicating they thought they would experience severe symptoms, severe symptoms requiring hospitalization (8.8%), or severe symptoms leading to death (2.3%).

### Knowledge

Participants were asked to respond to a series of true–false questions to assess their more general knowledge of COVID-19. The percentage of true, false, and unsure responses (with correct answers in bold font) can be seen in Table 3. Total general virus knowledge subscale scores ranged from 0 (1 respondent) to 19 (129 respondents), with a mean of 14.9 (*SD* = 2.8).

**TABLE 3:** Percentage of true, false, and unsure responses to general knowledge items, with correct answers in bold font.

	**True**	**False**	**Unsure**
Currently there is no vaccine to protect against COVID-19 coronavirus [T]	**95.0**	1.6	3.3
There is an effective medicine available for treating COVID-19 coronavirus [F]	5.1	**79.2**	15.6
There are ways to help slow the spread of COVID-19 coronavirus [T]	**89.7**	4.0	6.2
If COVID-19 coronavirus breaks out in Australia, it is likely that some people will have natural immunity to it [F]	29.5	**34.1**	36.3
The ordinary flu vaccine will protect me from COVID-19 coronavirus [F]	0.6	**92.7**	6.6
To date, no one in Australia has died from COVID-19 coronavirus [F*]	4.5	**91.8**	3.6
To date, no one in Australia who was infected with COVID-19 coronavirus passed it on to infect another person [F*]	4.1	**84.9**	10.9
There are other strains of coronaviruses that can infect humans, including those that cause the common cold [T]	**80.2**	4.7	15.0
The health effects of COVID-19 coronavirus appear to be more severe for people who already have a serious medical condition [T]	**97.7**	0.8	1.4
Antibiotics are an effective treatment for COVID-19 coronavirus [F]	3.4	**81.9**	14.5
Packages or letters from China can spread the virus [F]	6.8	**67.8**	25.3
Taking vitamin C or other vitamins will protect you from the COVID-19 coronavirus [F]	5.9	**74.1**	19.7
There is no evidence that vaccines against pneumonia will protect you against the COVID-19 coronavirus [T]	**67.0**	5.5	27.4
Regularly rinsing your nose with saline will protect you against the COVID-19 coronavirus [F]	2.7	**77.6**	19.6
There is no evidence that eating garlic will protect you against the COVID-19 coronavirus [T]	**82.6**	6.7	10.6
Putting sesame oil on your body will block the COVID-19 coronavirus from entering your body [F]	0.3	**95.4**	4.2
Hand dryers are effective in killing the COVID-19 coronavirus [F]	2.8	**80.2**	17.0
The virus was genetically engineered as part of a biological weapons program [F]	10.2	**57.6**	32.0
The virus was human-made and deliberately released [F]	10.2	**57.8**	31.9

*True during study design, false at data collection. Missing data from one to five respondents for each item; percentages do not always total 100.

Knowledge questions were also asked relating to most common symptoms and routes of transmission (see Table 4). Respondents were more accurate in recognizing the symptoms that have been linked with COVID-19 and less certain of whether the other symptoms (nausea, vomiting, and diarrhea) were indicative of illness. Symptoms knowledge subscale scores ranged from 0 to 7, with 32.6% of respondents correctly answering every item. The mean subscale score was 5.5 (*SD* = 1.4), indicating good recognition of the symptoms commonly mentioned in public health information provided to the Australian public at this time. Respondents typically recognized transmission routes associated with droplet spread but were less certain of whether the virus can also spread via air, water, or mosquitoes (evidence at the time indicated that these routes were unlikely). Transmission knowledge subscale scores ranged from 0 to 6, with a mean of 4.6 (*SD* = 1.0). Only 17.8% of respondents correctly answered every item.

**TABLE 4:** Percentage of yes, no, and unsure responses to symptoms and transmission knowledge items, with correct answers in bold font.

	**Yes (%)**	**No (%)**	**Unsure (%)**
**Symptoms**			
Fever	**97.5**	0.9	1.5
Cough	**96.7**	1.1	2.0
Sore throat	**86.1**	4.0	9.7
Shortness of breath	**90.4**	2.7	6.9
Nausea	20.9	**50.4**	28.4
Vomiting	8.5	**64.4**	26.8
Diarrhea	13.9	**66.0**	19.7
**Transmission**			
Droplets spread through coughing or sneezing	**98.8**	0.3	0.9
Surfaces recently touched by someone who is sick	**91.2**	3.1	5.7
Touching or shaking hands with a person who is sick	**94.8**	2.1	3.1
Airborne	56.1	**28.1**	15.8
Waterborne	8.0	**64.4**	27.3
Mosquitoes	1.7	**80.0**	18.0

Most respondents (79.7%) correctly identified that it was recommended (at the time) that people who were sick wear masks to stop them spreading the virus. In addition, 15.9% reported that “everyone”—both sick and healthy—should be wearing masks, and 1.3% responded that only healthy people should be wearing masks. Knowledge of the approximate mortality rate was good: 69.2% of respondents gave answers between 1% and 5%, which were deemed accurate. Percentage estimates ranged from 0 (0.5%) to 100 (0.3%), with a mean of 7.84% (*SD* = 12.31). A total COVID-19 knowledge score was calculated from responses to general, symptoms, and transmission subscales, as well as individual items about mask use and mortality. Scores ranged from 7 to 34 (out of a possible 34), with a mean of 26.48 (*SD* = 4.10).

### Health-Protective Behaviors

The percentage of respondents who reported having engaged in a range of distancing and hygiene behaviors during the past month can be seen in Table 5. Hygiene behaviors (handwashing, using hand sanitizing gel, and cleaning and disinfecting surfaces) were the most commonly reported behaviors. The number of behaviors endorsed was summed, and scores ranged from 0 (16%) to 13 (0.3%), with most (80.5%) respondents reporting five behaviors or fewer, with a mean score of 3.29 (*SD* = 2.89).

**TABLE 5:** Percentage of yes responses relating to health-protective behaviors during the past month.

	**Yes (%)**
Reduce or avoid going to work or university	6.3
Reduce or avoid using public transport	18.5
Reduce or avoid flying domestically	16.5
Reduce or avoid flying internationally	22.3
Reduce or avoid going to public events such as movies, sporting events, or concerts	25.4
Reduce or avoid going to hospitals or going to the doctor unless absolutely necessary	26.5
Reduce or avoid going into shops	18.2
Reduce or avoid staying in hotels, hostels, or Airbnb	13.8
Reduce or avoid sending your children to school or childcare	3.0
Clean or disinfect things you might touch (such as doorknobs or hard surfaces) more often than usual	39.1
Use sanitizing hand gel to clean your hands more often than usual	58.3
Wash your hands thoroughly more often than usual	76.3
Wear a face mask when going out in public	5.0

Four in five respondents indicated that they would definitely (60.4%) or probably (20.8%) get a vaccination if one became available. Only 12.3% reported being unsure, 3.7% said that they would probably not get the vaccine, and 2.8% said that they would definitely not get vaccinated.

### Predictors of Health-Protective Behaviors

Negative binomial regression with maximum likelihood estimation was conducted to assess the influence of information, perceived risk, and knowledge-related predictors on engagement with health-protective behaviors, while controlling for demographic factors and self-rated health. Negative binomial regression was chosen because it is appropriate for over-dispersed count data. The health-behavior outcome score is a count of the number of behaviors endorsed and is over-dispersed, as the variance of measures exceeds the mean score.

#### Demographic Predictors

To assess demographic differences in health-protective behaviors, each demographic predictor variable was entered individually into a separate negative binomial regression model. The mean (standard error) number of behaviors across demographics can be seen in Table 6. Demographic differences in health-protective behaviors were seen by gender (*p* < 0.001), state of residence (*p* = 0.002), age group (*p* = 0.001), and ethnicity (*p* < 0.001). Female respondents reported engaging in more health-protective behaviors than their male counterparts, and those in the youngest age group (18–29) engaged in fewer behaviors than older respondents. Behavior differences by ethnicity were also seen, with non-Caucasian respondents reporting more health-protective behaviors. Respondents from Queensland reported engaging in more behaviors than those from the category of NSW (reference category). There was not a significant effect of education level (*p* = 0.339).

**TABLE 6:** Demographic differences in the mean (SE) number of health-protective behaviors over the past month.

**Demographic variables**	**Health-protective behaviors M (SE)**
**Gender**	
Male (RC)	2.83 (0.12)
Female	3.45 (0.08)*
Non-binary, different identity, or prefer not to say	2.75 (0.43)
**State**	
New South Wales (RC)	3.15 (0.09)
Victoria	3.08 (0.16)
Queensland	3.86 (0.17)*
South Australia	2.70 (0.23)
Western Australia	3.40 (0.19)
Tasmania	3.47 (0.33)
Australian Capital Territory	3.38 (0.42)
Northern Territory	3.58 (0.73)
**Age group**	
18–29 (RC)	2.86 (0.12)
30–49	3.52 (0.11)*
50–59	3.25 (0.13)*
60+	3.32 (0.17)*
**Ethnicity**	
Caucasian (White/European; RC)	3.03 (0.07)
Australian Aboriginal or Torres Strait Islander	3.85 (0.25)*
Asian	4.74 (0.30)*
Other or prefer not to say	3.69 (0.24)*
**Highest education**	
High school only: completed (Year 12) or not completed (Year 11 or below; RC)	3.43 (0.13)
Trade certificate, diploma, or advance diploma	3.39 (0.13)
Bachelor’s degree	3.16 (0.12)
Graduate diploma, graduate certificate, or postgraduate degree	3.19 (0.12)

RC, reference category. *Significantly different from the RC at 0.05.

#### Psychological Predictors of Health-Protective Behaviors During the Past Month

To assess the influence of psychological predictors on engagement with health-protective behaviors, all relevant variables were entered into a single model (see Table 7), controlling for demographic variables and self-rated health. The Pearson Chi-Square Goodness of Fit statistic (1.084) indicated that the model fit the data well. The omnibus test results indicate that the model was a significant improvement over a null model, χ^2^ = 940.41 (*df* = 2), *p* < 0.001.

**TABLE 7:** Predictors of the number of health-protective behaviors during the past month.

	**95% Wald CI for Exp(B)**					
***Variable***	***B***	***SE***	***Exp(B)***	***Lower***	***Upper***	***p***
(Intercept)	−0.294	0.163	0.745	0.542	1.025	0.071
**Gender**						
Male (RC)	0	–	1	–	–	–
Female	0.091	0.040	1.096	1.013	1.185	0.022
Other	−0.066	0.143	0.936	0.707	1.238	0.642
**Age**						
18 to 29 (RC)	0	–	1	–	–	–
30 to 49	−0.037	0.044	0.964	0.883	1.051	0.404
50 to 59	−0.160	0.052	0.852	0.770	0.943	0.002
60 and over	−0.132	0.058	0.877	0.782	0.983	0.024
**Ethnicity**						
Caucasian (RC)	0	–	1	–	–	–
Australian Aboriginal and/or Torres Strait Islander	0.058	0.057	1.060	0.948	1.184	0.308
Asian	0.406	0.057	1.501	1.342	1.680	<0.001
Other/not stated	0.202	0.057	1.223	1.095	1.367	<0.001
**Education**						
High school only (RC)	0	–	1	–	–	–
Trade certificate or diploma	0.011	0.045	1.011	0.926	1.105	0.804
Bachelor’s degree	−0.046	0.047	0.955	0.871	1.048	0.333
Graduate diploma or postgraduate degree	−0.023	0.048	0.977	0.890	1.073	0.630
**State**						
New South Wales (RC)	0	–	1	–	–	–
Victoria	−0.018	0.049	0.982	0.892	1.081	0.716
Queensland	0.109	0.043	1.115	1.024	1.214	0.012
South Australia	−0.171	0.075	0.843	0.728	0.976	0.022
Western Australia	−0.027	0.051	0.973	0.881	1.075	0.594
Tasmania	0.035	0.082	1.036	0.882	1.216	0.670
Australian Capital Territory	−0.017	0.102	0.983	0.805	1.201	0.868
Northern Territory	−0.010	0.173	0.990	0.705	1.391	0.956
Self-rated health	−0.022	0.018	0.978	0.944	1.014	0.227
Exposure to media coverage	0.084	0.009	1.088	1.068	1.108	<0.001
Concern/worry about outbreak	0.295	0.020	1.343	1.292	1.395	<0.001
Science understands illness	−0.021	0.008	0.979	0.963	0.995	0.011
Confidence in government	−0.033	0.007	0.968	0.955	0.980	<0.001
Likelihood of outbreak	0.001	0.001	1.001	1.000	1.003	0.152
Severity of illness	0.046	0.018	1.047	1.010	1.085	0.012
Perceived effectiveness	0.003	0.001	1.003	1.001	1.004	0.001
Knowledge about illness	−0.010	0.004	0.990	0.982	0.998	0.018

*B, unstandardized coefficient; SE, standard error; Exp(B), exponentiated regression coefficient.*

More closely following media coverage, heightened worry or concern about an outbreak, higher perceived personal severity of COVID-19, and higher perceived effectiveness of health-protective behaviors were significant predictors of greater engagement with distancing and hygiene behaviors during the previous month. In contrast, stronger beliefs in scientific and medical understanding of the virus, confidence in government information, and higher COVID-19 knowledge scores predicted reduced engagement with health-protective behaviors.

### Predictors of Vaccination Intentions

Respondents were asked how likely they were to get vaccinated against COVID-19. This outcome did not approximate a normal distribution; thus, ordinal logistic regression was used to assess the influence of information, perceived risk, and knowledge-related predictors on vaccination intentions, while controlling for demographic factors and self-rated health.

#### Demographic Predictors

To assess demographic differences in vaccine intentions, each demographic predictor variable was entered individually into a separate ordinal logistic regression model. There were no demographic differences in vaccine intentions by gender (*p* = 0.429), state of residence (*p* = 0.832), ethnicity (*p* = 0.461), or level of education (*p* = 0.129). Respondents did differ in their vaccine intentions by age group (*p* = 0.019). Compared to the 60-plus age group, being in the 30–49 (*ExpB* = 0.662, 95% CI [0.503 to 0.871], *p* = 0.003) or 50–59 (*ExpB* = 0.695, 95% CI [0.515 to 0.938], *p* = 0.017) age group was associated with a lower likelihood of intending to get a vaccination (see Table 8 for percent of responses in each category by age group).

**TABLE 8:** Number and percent of respondents in each age group reporting that they would definitely not, would probably not, were unsure if they would, probably would, or definitely would get a COVID-19 vaccine, if available.

	**N (% of age group)**				
	**18–29**	**30–49**	**50–59**	**60+**	**Total**
Definitely not	6 (1.2)	24 (2.8)	17 (3.5)	13 (4.3)	60 (2.8)
Probably not	14 (2.9)	31 (3.6)	22 (4.5)	11 (3.6)	78 (3.7)
Unsure	44 (9.0)	118 (13.8)	76 (15.6)	28 (9.2)	266 (12.5)
Probably would	133 (27.2)	190 (22.2)	74 (15.2)	42 (13.9)	439 (20.6)
Definitely would	292 (59.7)	494 (57.6)	297 (61.1)	209 (69.0)	1,292 (60.5)

#### Psychological Predictors of Vaccination Intentions

Predictors entered into the full model were the same as in the previous analysis, with the addition of a dichotomous variable reflecting whether respondents had received a flu vaccine in the previous year or had not/were unsure. All variables were entered into a single model (see Table 9). The Pearson Chi-Square Goodness of Fit statistic (0.921) indicated good model fit. The omnibus test results indicate that the model is a significant improvement over a null model, χ^2^ = 557.23 (*df* = 28), *p* < 0.001.

**TABLE 9:** Predictors of likelihood of getting vaccinated against COVID-19 if a vaccine becomes available.

	**95% Wald CI for Exp(B)**					
***Variable***	***B***	***SE***	***Exp(B)***	***Lower***	***Upper***	***p***
**Gender**						
Male (RC)	0	–	1	–	–	–
Female	−0.451	0.119	0.637	0.505	0.803	<0.001
Other	0.197	0.397	1.218	0.560	2.650	0.619
**Age**						
18 to 29 (RC)	0	–	1	–	–	–
30 to 49	−0.722	0.131	0.486	0.375	0.628	<0.001
50 to 59	−0.866	0.155	0.420	0.310	0.570	<0.001
60 and over	−0.567	0.183	0.567	0.396	0.812	0.002
**Ethnicity**						
Caucasian (RC)	0	–	1	–	–	–
Australian Aboriginal and/or Torres Strait Islander	0.349	0.183	1.418	0.990	2.031	0.057
Asian	−0.210	0.181	0.810	0.569	1.155	0.245
Other/not stated	−0.049	0.175	0.952	0.676	1.343	0.781
**Education**						
High school only (RC)	0	–	1	–	–	–
Trade certificate or diploma	0.065	0.136	1.068	0.819	1.392	0.629
Bachelor’s degree	0.027	0.140	1.027	0.781	1.350	0.849
Graduate diploma or postgraduate degree	−0.192	0.142	0.825	0.625	1.089	0.175
**State**						
New South Wales (RC)	0	–	1	–	–	–
Victoria	−0.083	0.144	0.920	0.694	1.220	0.563
Queensland	−0.050	0.136	0.951	0.728	1.241	0.711
South Australia	−0.231	0.210	0.794	0.526	1.199	0.272
Western Australia	−0.183	0.152	0.833	0.618	1.122	0.229
Tasmania	0.391	0.272	1.479	0.868	2.519	0.150
Australian Capital Territory	−0.508	0.312	0.602	0.326	1.110	0.104
Northern Territory	−0.205	0.476	0.814	0.320	2.069	0.666
**Seasonal flu vaccine in past year**						
Yes (RC)	0	–	1	–	–	–
No or unsure	−1.719	0.102	0.179	0.147	0.219	<0.001
Self-rated health	−0.074	0.054	0.929	0.835	1.033	0.172
Exposure to media coverage	0.061	0.026	1.062	1.010	1.117	0.019
Concern/worry about outbreak	0.317	0.055	1.372	1.233	1.527	<0.001
Science understands illness	0.090	0.026	1.094	1.039	1.152	<0.001
Confidence in government	0.093	0.021	1.098	1.054	1.143	<0.001
Likelihood of infection	0.004	0.002	1.004	1.000	1.009	0.049
Severity of illness	0.108	0.061	1.115	0.989	1.256	0.076
Perceived effectiveness	−0.002	0.002	0.998	0.994	1.003	0.443
Knowledge about illness	0.050	0.012	1.051	1.027	1.076	<0.001

Having received a seasonal flu vaccine in the past year predicted increased intentions to get a COVID-19 vaccine if it becomes available. With regard to psychological predictors and in line with previous results, both increased exposure to media coverage and heightened worry or concern about the outbreak predicted increased vaccination intentions. In contrast to results relating to health-protective behaviors, perceptions of greater scientific and medical understanding of the virus, confidence in government information, and higher knowledge scores predicted greater vaccination intentions.

## Discussion

The results of the survey provide information on public knowledge, perceived risk and worry, and health-protective behaviors in the early period of the COVID-19 pandemic in Australia. A large proportion (two-thirds) of participants were at least moderately worried about the possibility of a widespread outbreak. These rates are commensurate with past pandemics such as SARS (Bults et al., 2011; Wheaton et al., 2012). Consistent with previous findings, higher worry about outbreaks was associated with greater health-protective behaviors (e.g., handwashing; Bults et al., 2011). Recent research from China indicates that engaging in hand hygiene and other health-protective behaviors was associated with reduced psychological impact of the COVID-19 outbreak, including lower stress and anxiety (Wang et al., 2020). These findings highlight the importance of encouraging the public to engage with such behaviors not only to reduce the risk of infection but also to reduce anxiety associated with COVID-19.

This study provided important insights into what participants *expected* in terms of how serious the symptoms of coronavirus would be, should they contract COVID-19. There is a clear discrepancy between respondents’ perceived severity of symptoms and current data on rates of asymptomatic infection. Only 0.3% of respondents believed that they would experience no symptoms. In contrast, emerging evidence from groups with widespread testing for the SARS-CoV-2 virus (e.g., cruise ships, repatriation flights, and overseas arrivals) indicates that between 2 and 8 out of every 10 infections may be asymptomatic (Day, 2020; Mizumoto et al., 2020; Nishiura et al., 2020). Despite being asymptomatic, those infected are still able to transmit the virus to others (Bai et al., 2020; Zou et al., 2020). In addition, people appear to be infectious and asymptomatic during the incubation period (Lauer et al., 2020). People commonly rely on symptoms to indicate illness and assume that the absence of symptoms means they are well (Diefenbach and Leventhal, 1996). Such assumptions in the COVID-19 pandemic could have serious consequences, in terms of both community transmission and reduced health-protective behaviors. Therefore, public health communication campaigns about COVID-19 need to address these misconceptions.

The results also provide insights into where Australian residents are seeking their information about COVID-19 and their level of knowledge about the virus and is transmission. While it was promising to see that 72% sourced information from official and government websites, mainstream news media was the most popular, and social media use was also high. The high usage of news media is concerning given the potential for alarming, sensationalist portrayals of the pandemic (Klemm et al., 2016). In addition, myths, rumors and misinformation can quickly spread online, particularly via social media (Vosoughi et al., 2018). Reliance on social media might have contributed to uncertainty around COVID-19, for example, about whether people have natural immunity and whether specific home remedies (garlic, vitamins, and rinsing noses with saline) help protect against coronavirus. It may also explain some uncertainty around whether the virus was human-made and deliberately released. Uncertainty and rapidly changing information may have contributed to increased worry about the virus (Han et al., 2006). These findings speak to the importance of distributing accurate health information about COVID-19 through a variety of sources (news, social media, and government websites) to reach the general population and correct misinformation.

Given the rapidly evolving situation with COVID-19 globally, the findings from this study may not be reflective of behaviors now that greater restrictions have been put in place and significant widespread messaging around social distancing, handwashing, and self-isolation has been disseminated. However, our findings provide insights into the demographic and psychological predictors of health-protective behaviors in the early stages of a pandemic disease outbreak. The most powerful predictors were demographic factors including age, female gender, and being of non-Caucasian ethnicity, as well as risk perceptions (greater worry about outbreak and perceived severity of illness) and higher media exposure. The effect of media exposure may be related to the provision of important health information about the pandemic. Although media exposure early in the outbreak appears to have facilitated health-protective behaviors, media fatigue—where people become desensitized to ongoing messaging—may reduce this effect as the pandemic continues (Collinson et al., 2015). Repeated media exposure may also lead to heightened stress and anxiety, which can have longer-term health effects, as well as contributing to excessive or misplaced health-protective behaviors such as presenting for diagnostic testing when actual risk of exposure is low (Garfin et al., 2020).

The results of this study shed light on how many participants plan to get a COVID-19 vaccine if available. Concern about the outbreak, greater media exposure, and higher knowledge predicted vaccination intentions. These findings are in line with previous research showing that concern and knowledge were associated with increased Ebola vaccine intentions (Petrie et al., 2016). In contrast to previous research, perceived likelihood and severity of infection were only marginally associated with intentions to get a vaccine (Weinstein et al., 2007; Bish and Michie, 2010). Previous research has typically focused on personal risk. In the case of COVID-19, the personal risk to most individuals is low, and behavior may be driven primarily by perceived risk to others, which was not assessed in the current study.

The current study is strengthened by a large sample size and a good representation of participants from different educational backgrounds. However, Caucasian women were overrepresented, as were those from NSW and those aged under 50 years. Participants were recruited through Facebook and as such are not representative of the general population. The pattern of results may not generalize to the broader population. To maximize convenience sampling, we used solely self-report measures, which may lead to biased effects. While the results of the regression analyses provide interesting starting points to identify the demographic and risk variables that predict health behaviors and vaccine intentions, they cannot establish causality and must be interpreted with caution. Given the large sample, the relationships between some of the significant predictors are likely to be small and may not be clinically meaningful.

The current results provide information on the Australian public responses to the COVID-19 pandemic, including information sources and engagement, knowledge, and perceived risk in the early stages of the outbreak in Australia, and their relationship with health-protective behaviors and vaccine intentions. The findings show that there was a critical mismatch between expected severity of symptoms versus data on how COVID-19 is experienced, which needs to be addressed in government education campaigns. Health-protective behavior was relatively low at the start of the outbreak, and these behaviors and vaccination intentions were consistently predicted by greater exposure to media and worry about outbreaks. Finally, our questions revealed significant uncertainty and misinformation, which needs to be corrected.

Without a vaccine currently available, encouraging widespread and sustained engagement with hygiene and distancing behaviors is critical to successfully manage the COVID-19 pandemic, flatten the curve of infections, and protect vulnerable individuals and overburdened healthcare systems. The results of the current study provide important insights into psychological and behavioral responses early in the outbreak of this novel coronavirus. The findings point to types of information that may be particularly effective and groups that may benefit from clear and targeted messaging to promote engagement with health-protective behaviors.

## Data Availability Statement

The raw data supporting the conclusions of this article will be made available by the authors, without undue reservation.

## Ethics Statement

The studies involving human participants were reviewed and approved by the UNSW HREAP-C (Behavioral Sciences) File 3309. The patients/participants provided their written informed consent to participate in this study.

## Author Contributions

KF and JN were responsible for the concept and design of the study, interpretation of results, and writing and critical review of the manuscript. KF was responsible for data collection and analysis. Both authors contributed to the article and approved the submitted version.

## Conflict of Interest

The authors declare that the research was conducted in the absence of any commercial or financial relationships that could be construed as a potential conflict of interest.
